# Genome-wide methylation profiling and copy number analysis in atypical fibroxanthomas and pleomorphic dermal sarcomas indicate a similar molecular phenotype

**DOI:** 10.1186/s13569-019-0113-6

**Published:** 2019-02-14

**Authors:** Christian Koelsche, Damian Stichel, Klaus G. Griewank, Daniel Schrimpf, David E. Reuss, Melanie Bewerunge-Hudler, Christian Vokuhl, Winand N. M. Dinjens, Iver Petersen, Michel Mittelbronn, Adrian Cuevas-Bourdier, Rolf Buslei, Stefan M. Pfister, Uta Flucke, Gunhild Mechtersheimer, Thomas Mentzel, Andreas von Deimling

**Affiliations:** 1 Department of General Pathology, Institute of Pathology, Heidelberg University Hospital, Im Neuenheimer Feld 224, 69120 Heidelberg, Baden-Württemberg Germany; 2 Department of Neuropathology, Institute of Pathology, Heidelberg University Hospital, Im Neuenheimer Feld 224, 69120 Heidelberg, Baden-Württemberg Germany; 3 Clinical Cooperation Unit Neuropathology, German Cancer Research Center (DKFZ), Heidelberg, Baden-Württemberg Germany; 4 German Cancer Consortium (DKTK), Core Center Heidelberg, Heidelberg, Baden-Württemberg Germany; 5 Department of Dermatology, University Hospital Essen, West German Cancer Center, University Duisburg-Essen and the German Cancer Consortium (DKTK), Essen, North Rhine-Westphalia Germany; 6 Dermatopathologie bei Mainz, Nieder-Olm, Rhineland-Palatinate Germany; 7 Genomics and Proteomics Core Facility, Microarray Unit, German Cancer Research Center (DKFZ), Heidelberg, Baden-Württemberg Germany; 8 Department of Pediatric Pathology, University Hospital of Schleswig-Holstein, Kiel, Schleswig-Holstein Germany; 9 Department of Pathology, Erasmus Medical Center, Rotterdam, The Netherlands; 10 Institute of Pathology, SRH Poliklinik Gera GmbH, Gera, Germany; 11 Luxembourg Centre of Neuropathology (LCNP), Luxembourg City, Luxembourg; 12 Laboratoire National de Santé (LNS), Dudelange, Luxembourg; 13 Luxembourg Centre for Systems Biomedicine (LCSB), University of Luxembourg, Luxembourg City, Luxembourg; 14 NORLUX Neuro-Oncology Laboratory, Luxembourg Institute of Health (LIH), Luxembourg City, Luxembourg; 15 Institute of Pathology, Sozialstiftung Bamberg, Bamberg, Germany; 16 Hopp Childrens Cancer Center at the NCT Heidelberg (KiTZ), Heidelberg, Germany; 17 Division of Pediatric Neurooncology, German Cancer Research Center (DKFZ), Heidelberg, Baden-Württemberg Germany; 18 Department of Pediatric Oncology, Hematology and Immunology, University of Heidelberg, Heidelberg, Baden-Württemberg Germany; 19 Department of Pathology, Radboud University Medical Center, Nijmegen, The Netherlands; 20 Dermatopathology Bodensee, Friedrichshafen, Baden-Württemberg Germany

**Keywords:** Pleomorphic dermal sarcoma, Atypical fibroxanthoma, Sarcomas, Melanomas, Carcinomas, Mimics, DNA methylation, Profiling

## Abstract

**Background:**

Atypical fibroxanthomas (AFX) and pleomorphic dermal sarcomas (PDS) are lesions of the skin with overlapping histologic features and unspecific molecular traits. PDS behaves aggressive compared to AFX. Thus, a precise delineation, although challenging in some instances, is relevant.

**Methods:**

We examined the value of DNA-methylation profiling and copy number analysis for separating these tumors. DNA-methylation data were generated from 17 AFX and 15 PDS using the Illumina EPIC array. These were compared with DNA-methylation data generated from 196 tumors encompassing potential histologic mimics like cutaneous squamous carcinomas (cSCC; n = 19), basal cell carcinomas (n = 10), melanoma metastases originating from the skin (n = 11), leiomyosarcomas (n = 11), angiosarcomas of the skin and soft tissue (n = 11), malignant peripheral nerve sheath tumors (n = 19), dermatofibrosarcomas protuberans (n = 13), extraskeletal myxoid chondrosarcomas (n = 9), myxoid liposarcomas (n = 14), schwannomas (n = 10), neurofibromas (n = 21), alveolar (n = 19) and embryonal (n = 17) rhabdomyosarcomas as well as undifferentiated pleomorphic sarcomas (n = 12).

**Results:**

DNA-methylation profiling did not separate AFX from PDS. The DNA-methylation profiles of the other cases, however, were distinct from AFX/PDS. They reliably assigned to subtype-specific DNA-methylation clusters, although overlap occurred between some AFX/PDS and cSCC. Copy number profiling revealed alterations in a similar frequency and distribution between AFX and PDS. They involved losses of 9p (22/32) and 13q (25/32). Gains frequently involved 8q (8/32). Notably, a homozygous deletion of *CDKN2A* was more frequent in PDS (6/15) than in AFX (2/17), whereas amplifications were non-recurrent and overall rare (5/32).

**Conclusions:**

Our findings support the concept that AFX and PDS belong to a common tumor spectrum. We could demonstrate the diagnostic value of DNA-methylation profiling to delineating AFX/PDS from potential mimics. However, the assessment of certain histologic features remains crucial for separating PDS from AFX.

**Electronic supplementary material:**

The online version of this article (10.1186/s13569-019-0113-6) contains supplementary material, which is available to authorized users.

## Background

Sarcomas of the skin and the adjacent soft tissue comprise a heterogeneous tumor group [1]. The classification of these tumors follows the lineage differentiation of tumor cells, which is predominantly assessed by their expression of lineage specific markers. However, in many cases an unambiguous subtype assignment by histologic and immunohistochemical means is not possible, and molecular analyses for establishing a final diagnosis is required [2]. Unfortunately, certain entities also lack unequivocal molecular traits, even if more sophisticated molecular approaches such as next generation sequencing are applied. Atypical fibroxanthomas (AFX) and pleomorphic dermal sarcomas (PDS) belong to the aforementioned group of ill-defined tumors and currently remain a diagnosis of exclusion [3].

AFX and PDS exhibit overlapping histologic features making a reliable distinction in many cases problematic [4]. The most important criterion in favor of the diagnosis PDS is an invading growth pattern into subdermal structures, which can be difficult to assess if small biopsies are provided for histopathological diagnostics [5]. Other diagnostic histologic features include necrosis, lymphovascular and perineural invasion. However, general features of anaplasia such as nuclear pleomorphism and atypical mitoses are common to both AFX and PDS [6, 7]. The distinction of AFX and PDS as different entities remains clinically important. AFX has an overall favorable biological behavior compared to the much higher potential for recurrence and metastasis in PDS [3, 6, 7]. Novel diagnostic approaches allowing a clear distinction of AFX and PDS would be of great value considering the steadily increasing incidence of skin cancers [8] and promising results of targeted therapies for certain dermal tumor subtypes [9, 10].

DNA-methylation profiling has evolved as a powerful method for determining cell differentiation. Array-based epigenotyping technologies nowadays enable large-scale high-throughput studies of DNA methylation patterns. The study of DNA-methylation in different cancers has already revealed molecular subgroups within known histologically defined tumor types [11–18] and led additionally to the discovery of new tumor types based on unique molecular features [19, 20]. Recently it has been shown to have great diagnostic capabilities determining the lineage of small blue round cell tumors not otherwise specified [21], cancers of unknown primary [22] and nervous system tumors [23].

AFX and PDS are generally believed to be of mesenchymal lineage, although a few studies have suggested an epithelial origin [24, 25]. Detailed DNA-methylation patterns in AFX and PDS have not been reported yet. We therefore performed genome-wide methylation profiling and copy number analysis of AFX, PDS and potential histologic mimics, with a focus on cutaneous squamous carcinomas (cSCC) and basal cell carcinomas (BCC) of the head and neck, alongside of melanomas and 11 soft tissue tumor entities.

## Materials and methods

### Sample selection

In total, 228 tumor specimens from different patients, all prototypical examples of their corresponding subtype, were included (Additional file 1: Table S1). AFX, PDS, cSCC and BCC were collected from the Dermatopathology Bodensee in Friedrichshafen (Germany) and the Department of Dermatology of the University Hospital in Essen (Germany). Melanomas and soft tissue tumors were collected from the Institute of Pathology of the University Hospital in Heidelberg (Germany), in Kiel (Germany), in Jena (Germany), in Nijmegen and in Rotterdam (both the Netherlands), from the Institute of Pathology in Bamberg (Germany) and from the Department of Pathology of the Laboratoire National de Santé (Luxembourg). Diagnoses were based on standard histopathological criteria in conjunction with immunohistochemical and molecular analyses according to the current WHO classification [1]. The methylation data of melanomas and some soft tissue tumors were published previously [12, 15, 21].

### DNA extraction

DNA was extracted from formalin-fixed and paraffin-embedded (FFPE) tumor tissue, thereby only using representative tumor tissue with highest available tumor content was chosen for genomic DNA isolation. The Maxwell^®^ 16FFPE Plus LEV DNA Kit was applied on the automated Maxwell device (Promega, Madison, WI, USA) according to the manufacturer’s instructions. Tumor DNAs had a total amount of > 100 ng and were suitable for the array-based DNA-methylation analysis.

### Genome-wide DNA-methylation data generation and pre-processing

The tumors were subjected to Illumina Infinium 450 k BeadChip or the successor EPIC/850 k BeadChip (Illumina, San Diego, USA) analysis at the Genomics and Proteomics Core Facility of the German Cancer Research Center (DKFZ) Heidelberg. DNA-methylation data were normalized by performing background correction and dye bias correction (shifting of negative control probe mean intensity to zero and scaling of normalization control probe mean intensity to 20,000, respectively). Probes targeting sex chromosomes, probes containing multiple single nucleotide polymorphisms and those that could not be uniquely mapped were removed. Probes were excluded if the predecessor Illumina Infinium 450 k BeadChip did not cover them, thereby making data generated by both 450 k and EPIC comparable for subsequent analyses. In total, 438,370 probes were kept for analysis.

### Unsupervised clustering, t-SNE analysis, cumulative copy number plotting and identification of differentially methylated regions

For unsupervised hierarchical clustering, we selected 10,000 probes that showed the highest median absolute deviation (MAD) across the beta values. Samples were hierarchically clustered using Euclidean distance and Ward’s linkage method. Methylation probes were reordered by hierarchical clustering using Euclidean distance and complete linkage. The unscaled methylation levels were shown in a heat map from unmethylated state (blue color) to methylated state (red color). For unsupervised 2D representation of pairwise sample correlations dimensionality reduction by t-distributed stochastic neighbor embedding (t-SNE) was performed using the 10,000 most variable probes, a perplexity of 20 and 2500 iterations. Copy-number assessment for segmental/entire chromosomal changes was done manually based on array data by a proprietary algorithm based on the R-package conumee after additional baseline correction (https://github.com/dstichel/conumee).

## Results

### Study cohort

Tumor samples from 61 patients with the histopathological diagnosis AFX (n = 17), PDS (n = 15), cSCC (n = 19) and BCC (n = 10) were analyzed together with 11 skin melanomas and 156 soft tissue tumors. The latter comprised 11 angiosarcomas, 13 dermatofibrosarcomas protuberans, 9 extraskeletal myxoid chondrosarcomas, 11 leiomyosarcomas, 14 myxoid liposarcomas, 19 malignant peripheral nerve sheath tumors, 21 neurofibromas, 19 alveolar and 17 embryonal rhabdomyosarcomas, 10 schwannomas and 12 undifferentiated pleomorphic sarcomas of the deep soft tissue. The median age was 81 years for AFX, 83 years for PDS, 79 years for cSCC and 77 years for BCC. The AFX, PDS, cSCC and BCC cohort consisted of 58 primary tumor samples, two recurrent samples and one case with an unknown status. The predominant side of occurrence was the head region (n = 46) followed by the neck (n = 9). AFX and PDS had a much higher incidence in male patients compared to cSCC and BCC. Clinical data are summarized in Table 1.

**Table 1:** Clinical features of atypical fibroxanthomas, pleomorphic dermal sarcomas, cutaneous squamous cell carcinomas and basal cell carcinomas

Category	AFX	PDS	cSCC	BCC
Group size (n)	17	15	19	10
Age median (range) [years]	81 (65–93)	83 (60–99)	79 (55–98)	77 (53–87)
Male/female	16/1	13/2	12/7	7/3
Tumor location	10 head, skin	14 head, skin	15 head, skin	7 head, skin
	7 neck, skin	1 unknown	2 hand, skin	2 trunk, skin
			1 neck, skin	1 neck, skin
			1 trunk, skin	

### Unsupervised genome-wide methylation profiling reveals distinct signatures in dermal sarcomas and histologic mimics

**Fig. 1:**
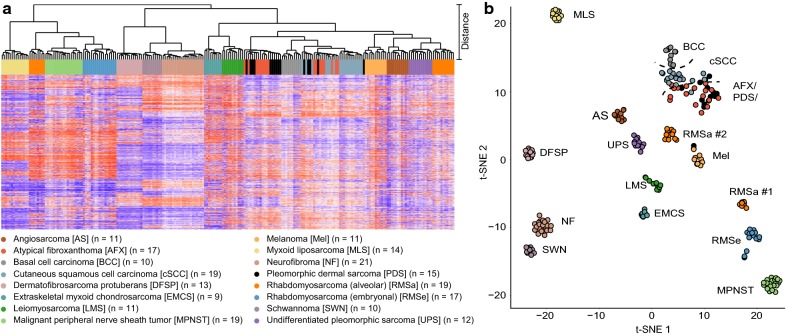
DNA-methylation profiling in atypical fibroxanthomas, pleomorphic dermal sarcomas and histologic mimics. Unsupervised hierarchical clustering (**a**) and t-Distributed Stochastic Neighbor Embedding (t-SNE) analysis (**b**) of DNA-methylation data from atypical fibroxanthomas (AFX), pleomorphic dermal sarcomas (PDS) and histologic mimics shows a close epigenetic relation to cutaneous squamous cell carcinomas (cSCC). This AFX/PDS/SCC methylation cluster clearly separated from the methylation clusters of other diagnostic mimics

Unsupervised hierarchical clustering and t-SNE analysis delineated tumors in methylation classes (Fig. 1), which also kept stable when varying the number of CpGs using for this analysis (data not shown). AFX and PDS were indistinguishable by clustering (Fig. 1a) and t-SNE analyses (Fig. 1b). cSCC and BCC grouped in close proximity to AFX and PDS. However, both formed homogeneous subgroups and therefore were distinct from these in both analyses, even though single cases overlapped (Fig. 1). Furthermore, we additionally analyzed 167 tumors encompassing 12 subtypes comprising different sarcoma entities and melanoma, which may mimic the phenotype of AFX and PDS. Each of these entities formed a subtype-specific methylation class. Interestingly, an obvious outlier case, initially diagnosed as PDS for lack of S100 and other melanoma specific staining (Additional file 2: Figure S1), repeatedly assigned to the methylation class of melanomas. Applying a targeted next generation sequencing panel the tumor demonstrated an activating *TERT* promoter mutation, a *HRAS* G12S mutation as well as a *BRAF* G466E mutation. Sequencing data are given in Additional file 3: Table S2.

### Cumulative copy-number profiling revealed overlapping patterns between atypical fibroxanthomas and pleomorphic dermal sarcomas

**Fig. 2:**
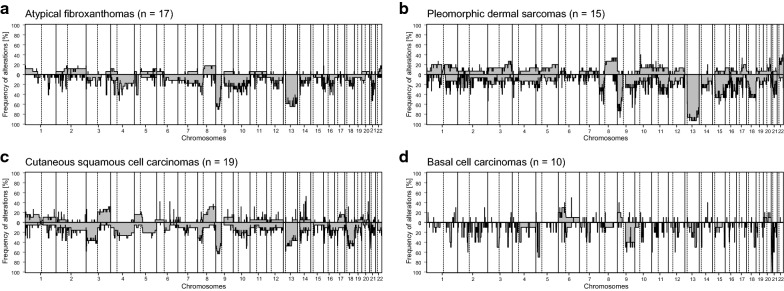
Cumulative copy number profiles. Frequency of copy number variations in 17 atypical fibroxanthomas (**a**), in 15 pleomorphic dermal sarcomas (**b**), in 19 cutaneous squamous cell carcinomas (**c**) and 10 basal cell carcinomas (**d**), assessed by automated aberrations profiling

We next generated copy number profiles derived from the DNA-methylation array data. AFX and PDS (Fig. 2a, b) revealed chromosomal imbalances that frequently involved losses of 9p (AFX 11/17; 65% vs. PDS 10/15; 66%) and 13q (AFX 11/17; 65% vs. PDS 14/15; 93%). A gain of chromosome arm 8q was slightly more frequent in PDS (5/15; 33%) compared to AFX (3/17; 18%). The homozygous deletion of the *CDKN2A* locus on 9p was more frequent in PDS (6/15; 40%) compared to AFX (2/17; 12%). Amplifications were rare in both AFX (3/15; 20%) and PDS (2/15; 13%). They were distributed in a non-recurrent pattern involving 5q21.3 (*FER*), 8p11.22-23 (*FGFR1*, *TACC1*) and 13q34 (*LAMP1*) in AFX, and 11q13.3 (*CCND1*) and 12q24.31 (*KNTC1*) in PDS (Additional file 4: Figure S2).

Copy number alterations in cSCC were distributed similarly to AFX and PDS (Fig. 2c). Chromosomal losses were frequently encountered on 3p (8/19; 42%), 13q (8/19; 42%) and 9p (12/19; 63%). Interestingly, the 19 cSCC demonstrated no homozygous deletions of the *CDKN2A* locus (9p). The most frequent gains involved 3q (4/19; 21%) and 8q (5/19; 26%). Amplifications were found in two cSCC involving *MYC* (8q24.21) and *CCND1* (11q13.3), respectively.

The copy number profiles of the 10 BCCs showed overall less frequent chromosomal gains and losses compared to AFX, PDS and SCC (Fig. 2d). Obvious amplifications and deletions were absent in BCC.

## Discussion

Our study demonstrates the predictive power of genome-wide methylation profiling in sarcomas of the skin (AFX/PDS) and their histologic mimics. Notably, all examined tumor subtypes exhibit specific epigenetic fingerprints with one exception. As expected, unsupervised clustering did not sort AFX and PDS into separate methylation groups. This finding is in line with the hypothesis that AFX and PDS are part of a common tumor spectrum with AFX potentially being a precursor lesion of PDS [3].

The concept of AFX and PDS comprising a single entity is supported by genetic studies [26, 27]. AFX and PDS carry similar, but yet unspecific patterns of *TP53* and *TERT* promoter mutations associated with UV-exposure such as observed in melanoma, cSCC and BCC [3, 26, 28, 29]. Recently, a next-generation sequencing based study on a considerable number of AFX and PDS expanded the overlapping mutational pattern to *NOTCH1*/*2* and *FAT1* [27]. However, only a single whole-exome study of AFX has been presented so far [30]. Thus, further whole-exome/genome studies with larger sample numbers of both AFX and PDS will be required to fully understand the genetic underpinnings of these tumors.

Copy-number aberrations were found in a comparable frequency and overlapping distribution in AFX and PDS. This is in concordance with previous studies showing recurrent copy number alterations mostly involving chromosome 8 and 9 [27, 31]. In addition, we found non-recurrent amplifications in 5/30 cases, which almost equally affected AFX and PDS. In contrast to our findings, a previous study detected amplifications only in PDS [31]. Hence, they suggested such markers for a tumor progression towards PDS. However, the study cohort was mainly composed of PDS (n = 24) with only three AFX cases for comparison.

Beside amplifications, we also noticed recurrent homozygous *CDKN2A* deletions in PDS (40%) and less frequently in AFX (12%). *CDKN2A* deletions have been recognized as an adverse prognostic marker in a number of tumors, i.e. in melanoma [32, 33]. Furthermore, a link between the susceptibility to checkpoint inhibitors and deletions of *CDKN2A* was discovered in some cell lines derived from SCC of the head and neck region (HNSCC) [34]. It remains to be determined whether this finding may be adapted to AFX/PDS and cSCC. If validated in further studies, *CDKN2A* status might prove as a valuable biomarker in AFX and PDS that might open new therapeutic avenues in a substantial portion of patients suffering from this disease.

Our study does not provide a final decision on the ongoing debate regarding the histogenesis of AFX and PDS. Many experts assume that AFX and PDS derive from a mesenchymal origin [1, 4], whereas others suggest that AFX may derive from an epithelial origin [30, 35]. This theory was initially introduced by older studies describing clinicopathological similarities between AFX and cSCC with a sarcomatoid dedifferentiation [36, 37]. AFX similar to cSCC and BCC frequently shows an association with actinic skin damage and a close proximity between the epithelium and the neoplastic spindle cell population, however without an epithelial dysplasia or carcinoma in situ component, which are both features and arguments for the diagnosis of a cutaneous spindle cell carcinoma with loss of keratin expression [1, 38, 39]. Although we noticed a separation of BCC from cSCC and AFX/PDS by epigenetic profiling and also a remarkable delineation between AFX/PDS and cSCC, DNA-methylation profiles of individual AFX, PDS and cSCC were overlapping. Thus, the DNA-methylation analysis primarily recapitulated the morphology of BCC, cSCC and AFX/PDS, which is usually quite distinct.

Correctly distinguishing AFX/PDS from other tumors is critical to allocate affected patients to the correct type of treatment and follow-up protocols. The current diagnosis of AFX/PDS based primarily on lack of expression of certain lineage markers. However, there is a constant risk that tumors of other lineages may have lost expression of diagnostically relevant markers due to dedifferentiation and then may be misclassified as AFX/PDS. For certain entities, such as the illustrated example where methylation and gene mutation signatures argue for a melanoma, misclassification could have significant consequences for the patient [40].

Therefore, it would seem prudent to perform molecular testing of cutaneous neoplasms when making a definitive diagnosis is not possible based on histomorphological and immunohistochemical assessment alone.

## Conclusion

Our study demonstrates a proof of concept that DNA-methylation may be a valuable aid in routine diagnostics of skin tumors posing a diagnostic challenge with conventional analytic methods. Our data support the concept that AFX and PDS are histologically and molecularly closely related and probably belong to a common tumor spectrum. We observed a *CDKN2A* deletion in AFX (12%) and PDS (40%), which may represent a potential biomarker, if validated in future studies. Copy number analysis and DNA methylation profiling can aid in distinguishing AFX/PDS from other histologic mimics, even though these analyses alone cannot reliably distinguish AFX from PDS. The assessment of histopathological features such as subcutaneous involvement, necrosis, and lymphovascular or perineural invasion still remain critical in differentiating PDS from AFX.

## Additional files

**Additional file 1: Table S1.** Clinical data. ID—internal identifier, Dx—diagnosis, Samp—sample, AS—angiosarcoma, AFX—atypical fibroxanthoma, BCC—basal cell carcinoma, cSCC—cutaneous squamous cell carcinoma, DFSP—dermatofibrosarcoma protuberans, EMCS—extraskeletal myxoid chondrosarcoma, LMS—leiomyosarcoma, MPNST—malignant peripheral nerve sheath tumor, Mel—melanoma, MLS—myxoid liposarcoma, NF—neurofibroma, PDS—pleomorphic dermal sarcoma, RMS—rhabdomyosarcoma, SWN—schwannoma, UPS—undifferentiated pleomorphic sarcoma, P—primary, R—recurrence, Me—metastasis, U—unknown, f—female, m—male.

**Additional file 2: Figure S1.** Histologic and immunohistochemical features of a pleomorphic dermal sarcoma with a DNA-methylation pattern resembling melanoma. This highly cellular tumor (ID 101138) with brisk mitotic activity (green arrows) predominantly presented with a polygonal to spindle-shape appearance and a fascicular growth pattern (a). In a circumscribed area the tumor cells were epithelioid (b). Adjacent subcutaneous fat tissue was infiltrated (c) and vascular invasion was observed (d). Parts of the tumor were necrotic (e). The tumor cells did not bind S100 specific antibody, whereas peripheral nerve and few histiocytes were positive (f). The tumor cells were negative for nuclear SOX10 expression with peripheral nerve as positive internal control (g), negative for HMB45 (h) and MelanA (i) protein expression. Scale-bars equal 100 µm.

**Additional file 3: Table S2.** List of gene mutations revealed by panel sequencing in a pleomorphic dermal sarcoma with discordant DNA-methylation profile.

**Additional file 4: Figure S2.** Copy number profiles of the three atypical fibroxanthomas and the two pleomorphic dermal sarcomas carrying gene amplifications.

## Abbreviations

MAD: median absolute deviation
t-SNE: t-distributed stochastic neighbor embedding
DKFZ: German Cancer Research Center
FFPE: formalin-fixed and paraffin-embedded
ID: internal identifier
Dx: diagnosis
Samp: sample
AS: angiosarcoma
AFX: atypical fibroxanthoma
BCC: basal cell carcinoma
cSCC: cutaneous squamous cell carcinoma
DFSP: dermatofibrosarcoma protuberans
EMCS: extraskeletal myxoid chondrosarcoma
LMS: leiomyosarcoma
MPNST: malignant peripheral nerve sheath tumor
Mel: melanoma
MLS: myxoid liposarcoma
NF: neurofibroma
PDS: pleomorphic dermal sarcoma
RMS: rhabdomyosarcoma
SWN: schwannoma
UPS: undifferentiated pleomorphic sarcoma
P: primary
R: recurrence
Me: metastasis
U: unknown
f: female
m: male
