# Supplementary figures and images for: Genome-wide methylation profiling and copy number analysis in atypical fibroxanthomas and pleomorphic dermal sarcomas indicate a similar molecular phenotype
Source: Clin Sarcoma Res. 2019 Feb 14;9:2. doi: 10.1186/s13569-019-0113-6 (PMC6375211; doi:10.1186/s13569-019-0113-6)

a HE

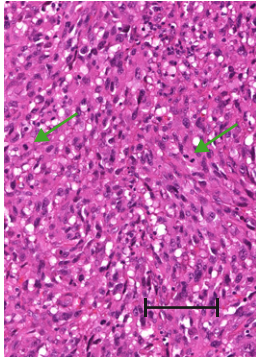

b HE

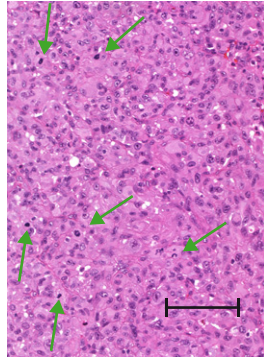

c HE

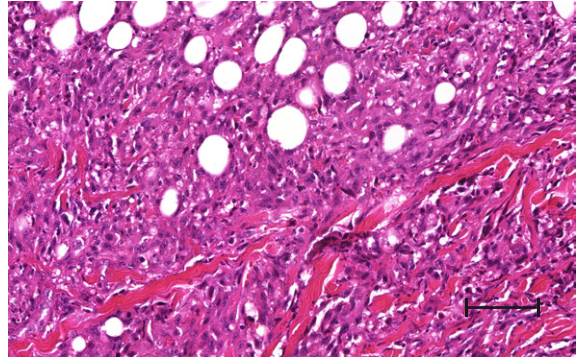

d HE

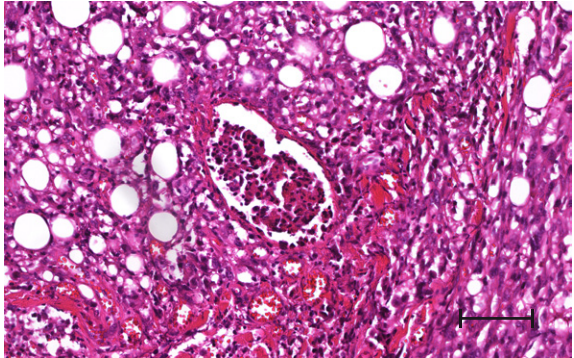

e HE

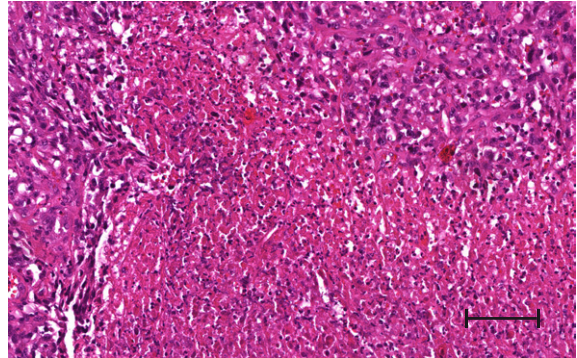

f S100

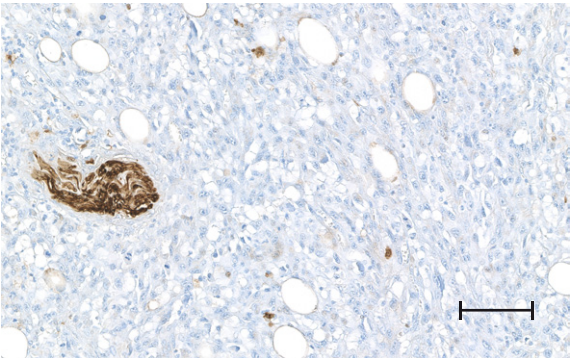

g SOX10

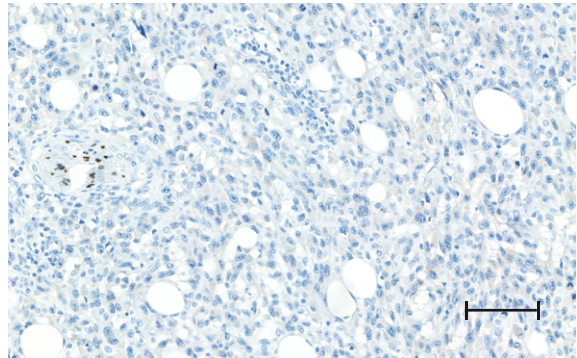

h HMB45

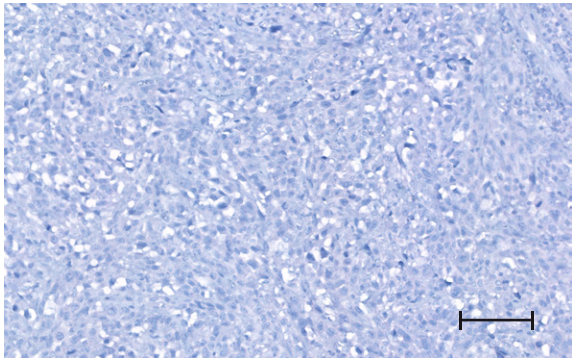

i MelanA

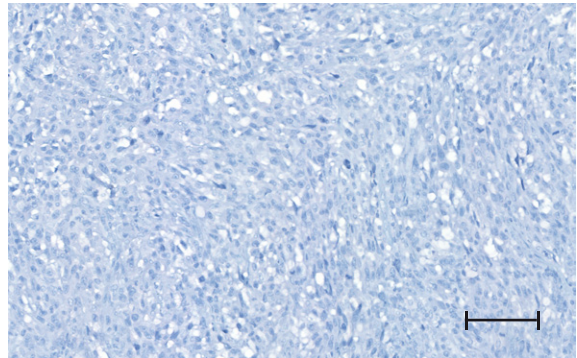

Supplement: Supplementary file 2 — Additional file 2: Figure S1. Histologic and immunohistochemical features of a pleomorphic dermal sarcoma with a DNA-methylation pattern resembling melanoma. This highly cellular tumor (ID 101138) with brisk mitotic activity (green arrows) predominantly presented with a polygonal to spindle-shape appearance and a fascicular growth pattern (a). In a circumscribed area the tumor cells were epithelioid (b). Adjacent subcutaneous fat tissue was infiltrated (c) and vascular invasion was observed (d). Parts of the tumor were necrotic (e). The tumor cells did not bind S100 specific antibody, whereas peripheral nerve and few histiocytes were positive (f). The tumor cells were negative for nuclear SOX10 expression with peripheral nerve as positive internal control (g), negative for HMB45 (h) and MelanA (i) protein expression. Scale-bars equal 100 µm. [file 13569_2019_113_MOESM2_ESM.pdf]
