# Supplementary material for: Genome-wide methylation profiling and copy number analysis in atypical fibroxanthomas and pleomorphic dermal sarcomas indicate a similar molecular phenotype
Source: Clin Sarcoma Res. 2019 Feb 14;9:2. doi: 10.1186/s13569-019-0113-6 (PMC6375211; doi:10.1186/s13569-019-0113-6)

AFX 101176 with 5q21.3 amplification

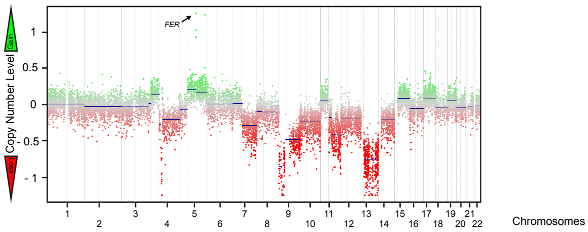

AFX 101174with 8p11.22-23 amplification

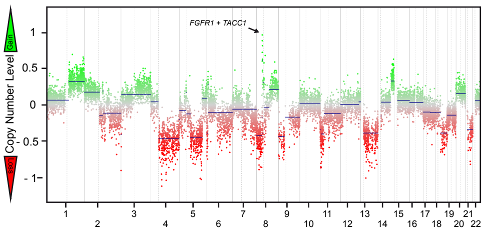

AFX 101166 with 13q34 amplification

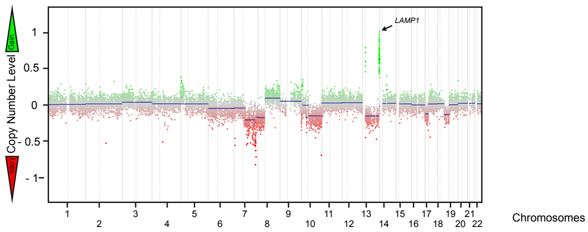

PDS 101152 with 11q13.3 amplification

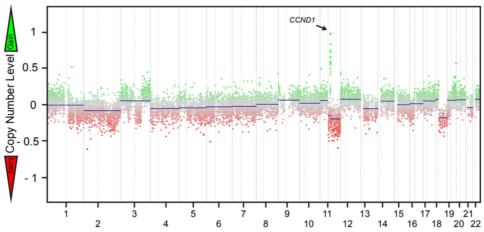

PDS 101158 with 12q24.31 amplification

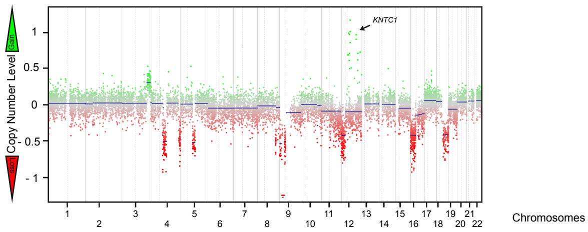

Supplement: Supplementary file 4 — Additional file 4: Figure S2. Copy number profiles of the three atypical fibroxanthomas and the two pleomorphic dermal sarcomas carrying gene amplifications. [file 13569_2019_113_MOESM4_ESM.pdf]
